# Recommendations for Keeping Parks and Green Space Accessible for Mental and Physical Health During COVID-19 and Other Pandemics

**DOI:** 10.5888/pcd17.200204

**Published:** 2020-07-09

**Authors:** Sandy J. Slater, Richard W. Christiana, Jeanette Gustat

**Affiliations:** 1 Department of Pharmaceutical Sciences and Administration, Concordia University, Mequon, Wisconsin; 2 Physical Activity Policy Research and Evaluation Network, Parks and Green Space Work Group, Centers for Disease Control and Prevention, Atlanta, Georgia; 3 Department of Health and Exercise Science, Appalachian State University, Boone, North Carolina; 4 School of Public Health and Tropical Medicine, Tulane University, New Orleans, Louisiana

## Abstract

The importance of engaging in any type of physical activity regularly, for both physical and mental health, is well established, and may be particularly beneficial in protecting the body and limiting the damage caused by the coronavirus disease 2019 (COVID-19). Exposure to nature or green space also has positive physical and mental health benefits. Closures of parks and green spaces during the COVID-19 pandemic has limited the options for physical activity and may affect vulnerable populations more than others. We provide both short-term and long-term recommendations to encourage access to green space for people while allowing for physical distancing.

**Summary**

**What is already known on this topic?**

Engaging in regular physical activity and having access to nature or green space are beneficial for physical and mental health.

**What is added by this report?**

Shelter-in-place and safer-at-home orders limit access to parks and green space for many people. We propose some short- and long-term solutions that can provide access to green space, while allowing for physical distancing.

**What are the implications for public health practice?**

These solutions may be useful and informative for cities, states, and countries around the globe as they implement policies to address the coronavirus disease 2019 pandemic, which can lead to healthier communities and populations.

## Introduction

The importance of engaging in any type of physical activity regularly, including exercising for both physical and mental health, is well established and, more important, may be particularly beneficial in protecting the body and limiting the damage caused by the coronavirus disease 2019 (COVID-19) (1). Engaging in regular physical activity is also protective against poor cardiovascular health, obesity, hypertension, and diabetes, which are shown as risk factors for COVID-19 (1). Exposure to nature or green space also has positive physical and mental health benefits, including lower rates of heart disease, stroke, obesity, stress, and depression (2). In fact, exposure to green space, even in a limited setting (eg, residential city streets in urban areas), is just as beneficial for health as that of visiting a natural setting or large public park (3).

In March 2020, the majority of United States governors issued shelter-in-place orders (4). Collectively, these orders severely restricted movements of individuals across the nation (4). These orders resulted in the closure of primary, secondary, and post-secondary schools; local fitness, physical activity, and recreational facilities; sports clubs; and non-essential businesses. Yet public health entities, such as the American Public Health Association and the Centers for Disease Control and Prevention, have stressed the importance of staying physically active while sheltering in place during COVID-19, which includes visiting parks and green space (5). With the closure of schools, fitness facilities, and other community places for recreation, local streets, parks, trails, and open green spaces are the only places available for physical activity outside of the home environment (6). However, many public parks and green spaces were also closed because of concerns about social distancing, and most state and local shelter-in-place orders allow only limited use of parks and green space (6). For example, people may access parks and green space near their homes, but playgrounds and equipment, sports courts, and trails are likely closed to the public. These restrictions might contribute to increased adverse physical and mental health outcomes for a substantial portion of the population, particularly those in urban settings, which, in turn, may be negatively associated with how well people can fight COVID-19 (1,7). The latest research shows that people must have sustained contact of 10 minutes or more (7) and be less than 6 feet (8) from others to be most susceptible to contracting COVID-19. If park, trail, and playground patrons remain appropriately physically distant, do not engage in lengthy conversations with nonhousehold members, and wear a protective face mask, their risk of contracting COVID-19 by exercising outdoors is low, making parks and green spaces safe places to be physically active during a pandemic (9). Being quarantined is associated with poor mental health outcomes (10), but maintaining access to parks and green space could counteract these negative effects.

Shelter-in-place orders limit physical activity options for everyone but have a greater effect on vulnerable populations (6). For example, racial minorities, such as African Americans, contract COVID-19 at higher rates than non-Hispanic whites and are disproportionately dying from the disease (11,12). These same populations tend to live in dense urban areas (13,14) with limited green space, and often in multiunit housing (11,12). Urban areas also have a greater likelihood of park deserts (ie, a defined geographic area that does not have a park present and accessible for use), or only small parks with limited features (15). These small parks are more likely to be restricted from public use during statewide shelter-in-place orders because of their size and might be dominated by play structures and banned from use (16). Communities lacking parks might need to explore alternative solutions for physical activity in outdoor public spaces. Urban and minority populations might also be reliant on public transit, which has been restricted to use for work or other essential needs (eg, purchasing groceries). Use of public transit for leisure activities (eg, visiting parks or other green spaces) is not recommended in many areas. Shelter-in-place orders might exacerbate inequities for people to access parks or green spaces if they do not live near them. Although the recommendations we provide can apply to a wide variety of populations in urban, suburban, and rural settings, they may be particularly relevant for minority populations in urban settings.

## Recommended Strategies to Address Parks and Green Space Accessibility

A recent article highlights ways to be physically active in the home, but these recommendations lack suggestions regarding access to green space (17). Exercising at home might be adequate and feasible for certain segments of the population, but many people live in homes with limited space or other factors that negatively affect health. The relationship between housing conditions and health is well established (18). Although most states are partially or fully lifting shelter-in-place orders, maintaining some physical distancing (19) is recommended until a vaccine is developed or until adequate immunity is realized within the population. Reintroduction of shelter-in-place orders might be necessary in response to an increase in COVID-19 cases or for future communicable disease outbreaks.

In this commentary, we propose some solutions that can be implemented, now or in the future, to provide access to green space while allowing physical distancing. Our recommendations are not necessarily new or novel ideas. Several of the strategies and policy recommendations proposed here have been advocated for various public health sectors for more than a decade (20–24). The COVID-19 pandemic has highlighted these long-known deficiencies in walking, biking, and recreational infrastructure (25,26) that contribute to health disparities. We hope that some of the solutions we offer can be useful and informative for cities, states, and countries around the globe as they implement their own policies to address the COVID-19 pandemic. Ours is not a comprehensive list nor a list that can or should be implemented in all places; it is meant to be a starting point for a conversation between national, state, and local governments, parks and recreation departments, other nonprofit organizations (eg, National Recreation and Park Association, Trust for Public Land, sports leagues, philanthropic park partners), and researchers.

### Short-Term Recommendations

**Keep parks open**
For both urban and rural areas, state and local parks with trails and open green space should remain open. Modifications in scheduling might be needed to help control the number of visitors at one time and allow for appropriate physical distancing.
  - This could include structured schedules, time slots, or sign-up sheets either in person or online for smaller parks, or monitoring by park staff in larger parks.
  - Staff from other departments may be needed to help ensure physical distancing guidance and that other rules are followed.
  - Park visits and access to other green spaces could be proactively prioritized and formally organized for vulnerable populations.
  - For parks with fees, fees could be adjusted on the basis of need. People who receive SNAP (Supplemental Nutrition Assistance Program) or Medicaid could have a reduced fee. Caution should be taken in terms of waiving fees for everyone as this might lead to a large increase in park visitation and crowding, as was seen in some parks early during the COVID-19 pandemic.
  - Evaluate policies that change schedules and modify fees, to determine best practices in balancing expanded access with strategies to control the number of visitors.
**Modify policies on the use of public transit **
During shelter-in-place orders, maintain transit routes to parks and green space. Allow riders to use transit to access parks and green space. Require public transit users to wear masks or face coverings and maintain physical distance. Public transit access to essential businesses and services (eg, healthcare facilities, grocery stores, and child care centers) must be balanced with access to parks and green space.
**Adopt Open Streets or Slow Streets initiatives**
Particularly in urban areas, such initiatives will allow closure of certain streets to vehicle traffic during specific days and times so that pedestrians and cyclists have more space to move. Some cities that have permanently adopted these initiatives could be evaluated to determine the impact of these initiatives (27). Streets with greenery, plants, or other natural features can be prioritized for these initiatives, given the positive association between public green space and mental health (28–30). To increase access to parks and green spaces, streets surrounding or connecting them could be designated as Open or Slow Streets.
**Adopt consistent messaging**
Consult communication resources for use of parks, such as the Centers for Disease Control and Prevention (https://www.cdc.gov/coronavirus/2019-ncov/daily-life-coping/visitors.html) (5) and the National Recreation and Park Association (https://www.nrpa.org/our-work/Three-Pillars/health-wellness/coronavirus-disease-2019/).
  - Messages should be targeted to the specific population, especially vulnerable and marginalized populations. Consider messages in multiple languages and the use of pictograms or diagrams.
  - Consideration should be given to the appropriate messengers and format for delivery.
  - Emphasis should be placed on maintaining appropriate physical distancing, not social isolation (19).

### Long-Term Recommendations

**Create built environments for all users**
Infrastructure plans should include policies and plans for creating healthy environments, such as Complete Streets, Safe Routes to Parks, Safe Routes to Schools, and mixed-use policies (20,24). Plans should also intentionally include green space and public spaces for leisure and recreation.
  - Ensure that including green space is prioritized on streets in neighborhoods that lack them. Municipalities should review local design guidelines and zoning codes to ensure they include provisions for greenscapes, green streets, sidewalk planters, or other greening strategies.
  - Consider access for all users through various approaches. Install protected bicycle lanes (ie, provide physical barriers between cars and bicyclists) or pedestrian connections to local trails, paths, parks, and green spaces. Increase parking for bicycles at parks and green spaces. Ensure public spaces comply with the Americans with Disabilities Act regulations. Engage with community members to explore availability, accessibility, and quality issues that are important to the community.
  - Plan for maintenance and regular improvements of green spaces and parks.
**Consider where to locate parks and green spaces**
Ensure that quality parks and green spaces are located in close proximity to people, regardless of where they live.
**Conduct ongoing monitoring and evaluation**
To ensure that any strategies implemented work in the expected ways, plan for ongoing monitoring and evaluation. This should include examining any unintended consequences, such as decreased sanitary conditions, litter, substandard bathroom facilities, and increased crime.
  - Evaluation should include the impact of strategies on mental and physical health.
  - Create a national open platform for policy makers and researchers to share evidence-based strategies. Learning from the successes and mistakes of implementing these strategies is vital during this unprecedented situation.

These recommendations can apply to all settings, including rural main streets and suburban areas, but they might be particularly important for urban areas. We have highlighted several advantages to keeping parks open during a pandemic. Careful consideration of potential disadvantages is also essential. For example, with most public settings inaccessible, keeping parks and green space open could lead to overcrowding, making it difficult to maintain physical distance and resulting in increasing the spread of disease. Significant increases in park visitors could also add strains to local budgets and staff members (ie, maintenance and cleaning responsibilities might increase). Strains might also increase risk of illness or other unintended consequences to staff. Local communities might not have access to the resources needed to appropriately staff and maintain parks during a pandemic. Finally, less is known about how COVD-19 spreads in outdoor settings. The virus might be susceptible to sunlight (31). If COVID-19 transmission risk is lower outdoors, the efficacy of adhering to physical distancing guidelines (8) and avoiding prolonged close proximity to other people (7) might be increased. More studies are needed to evaluate the likelihood of contracting the disease while exercising outdoors.

## Implications for Public Health

The COVID-19 pandemic has illuminated underlying disparities in access to parks and green space for underserved and vulnerable populations. Building a stronger infrastructure of neighborhood parks and green space throughout the country will help limit the impact of future public health disasters. Before and during a pandemic, national, state, and local policy makers, urban planners, and governments should thoughtfully consider what is appropriate and important for overall population health and how best to implement some of the recommendations proposed while maintaining appropriate physical distancing in public spaces. Access to parks and green space is vitally important for the health and well-being of individuals, and it will lead to healthier populations.
